# Explainable AI-Based Feature Selection Approaches for Raman Spectroscopy

**DOI:** 10.3390/diagnostics15162063

**Published:** 2025-08-18

**Authors:** Nicola Rossberg, Rekha Gautam, Katarzyna Komolibus, Barry O’Sullivan, Andrea Visentin

**Affiliations:** 1 Taighde Éireann—Research Ireland Center for Research Training in Artificial Intelligence, University College Cork, College Road, T12 K8AF Cork, Ireland; osullivan.barry@ucc.ie (B.O.); andrea.visentin@ucc.ie (A.V.); 2 School of Computer Science and Information Technology, University College Cork, College Road, T12 K8AF Cork, Ireland; 3 Tyndall National Institute, Lee Maltings Complex Dyke Parade, T12 R5CP Cork, Ireland; rekha.gautam@tyndall.ie (R.G.); katarzyna.komolibus@tyndall.ie (K.K.); 4 Insight Center for Data Analytics, University College Cork, College Road, T12 K8AF Cork, Ireland

**Keywords:** Explainable AI, Biophotonics, machine learning, Tissue Classification, feature selection, Raman spectroscopy

## Abstract

**Background**: Raman Spectroscopy is a non-invasive technique capable of characterising tissue constituents and detecting conditions such as cancer with high accuracy. Machine learning techniques can automate this task and discover relevant data patterns. However, the high-dimensional, multicollinear nature of Raman data makes their deployment and explainability challenging. A model’s transparency and ability to explain decision pathways have become crucial for medical integration. Consequently, an effective method of feature-reduction while minimising information loss is sought. **Methods**: Two new feature selection methods for Raman spectroscopy are introduced. These methods are based on explainable deep learning approaches, considering Convolutional Neural Networks and Transformers. Their features are extracted using GradCam and attention scores, respectively. The performance of the extracted features is compared to established feature selection approaches across four classifiers and three datasets. **Results**: We compared the proposed method against established feature selection approaches over three real-world datasets and different compression levels. Comparable accuracy levels were obtained using only 10% of features. Model-based approaches are the most accurate. Using Convolutional Neural Networks and Random Forest-assigned feature importance performs best when maintaining between 5–20% of features, while LinearSVC with L1 penalisation leads to higher accuracy when selecting only 1% of them. The proposed Convolutional Neural Networks-based GradCam approach has the highest average accuracy. **Conclusions**: No approach is found to perform best in all scenarios, suggesting that multiple alternatives should be assessed in each application.

## 1. Introduction

**Figure 1:**
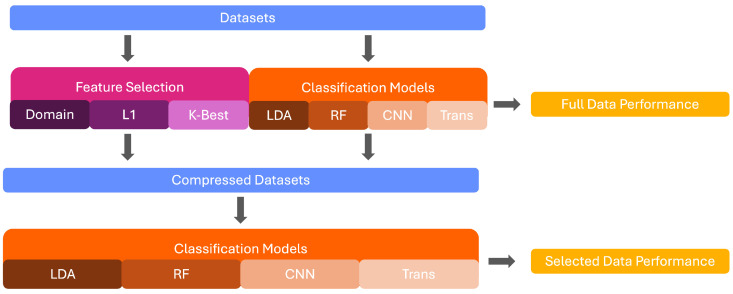
Graphical Depiction of this study’s workflow.

Raman spectroscopy can successfully characterise the molecular composition of various materials [1,2]. It has been found to provide high diagnostic accuracy for the identification and grading of various illnesses and is favoured due to its non-invasive and non-destructive nature [3,4,5]. However, the high-dimensional and frequently noisy nature of Raman signals makes it difficult to distinguish spectra through visual inspection only. The use of machine learning algorithms, which are adaptable at extracting patterns and performing successful classification on their basis, has thus been established as a favourable possibility for the classification of Raman spectra [6,7]. Some of the most commonly implemented models include Support Vector Machine (SVM) [8,9], Linear Discriminant Analysis (LDA) [10,11], and Random Forest [12,13]. However, the deployment of these models in the medical context is dependent on their ability to explain decision pathways to prevent bias and promote the trust of patients and practitioners alike [14]. The existing implementations of explainability measures in the field of machine learning rely on the identification of classification-relevant features. This is problematic in the domain of Raman spectroscopy due to the highly correlated nature of the signals. Wavenumbers marked as important by explainability techniques may only partially represent the underlying class or be a result of co-variation with the relevant wavenumbers. As such, it is important to reduce the number of features before the deployment of explainability to improve the precision of the implemented techniques. In this paper, we implement and compare seven feature selection techniques across three medical Raman Spectroscopy datasets and compare the performance of four classifiers across various degrees of data compression. This provides insights to inform methodological choices for feature selection and classifier choice in future studies. The workflow of this study is shown in Figure 1.

## 2. Literature Review

The reduction of features is feasible through both feature selection and extraction techniques. While feature extraction generates new features by combining existing variables and creating meta-features, feature selection filters the available variables to retain the most important ones [15]. Feature extraction, such as principal component analysis, can compensate for the high multicollinearity of Raman data by creating new variables based on covariance. However, two problems are associated with this. First, the technique assumes that high variance is representative of important information, which is a false equivalent and can hence obscure rather than capture important patterns [16]. Second, the creation of meta-features limits model interpretability and explainability as the classification-relevant features cannot be traced due to the transformation of the input space during the extraction process [17]. On the other hand, feature selection is a filter-based method that selects the most important features for retention. Feature selection is advantageous for several reasons. First, different from feature extraction, it maintains the connection of the selected features and underlying biological components, allowing interpretability in a reduced feature space [17]. Feature selection can also be computationally advantageous as it does not require the computation of new features, easing its implementation. Furthermore, depending on the chosen technique, feature selection can account for the complex nature of Raman data, selecting relevant variables in the presence of correlation and noise. Hence, feature selection offers several advantages over feature extraction in this context.

The implementation of feature selection in conjunction with opaque machine learning models has been repeatedly studied in the field of Raman spectroscopy and has been found to allow analysis of classification-relevant components while maintaining high classification accuracy [18,19,20]. In the study by [21], the authors utilise a long-short term memory network for multi-scale sequential feature selection in Raman Data. This yields the advantage of capturing long-term dependencies between features, which is suitable for the high-dimensional and intercorrelated nature of Raman signals. The authors evaluate the approach across three medical datasets and report accuracies between 76.3–97.9% depending on the dataset, emphasising the promise of this technique. Notably, the classification accuracy improves after feature selection, emphasising the promise of the technique for both performance and explainability. A different study by [22] proposes a new feature selection technique based on Fisher feature selection and support vector machines. The authors conduct supervised classification in tandem with feature selection based on a user-defined Fisher criterion, preventing overfitting and identifying relevant features. This methodology is deployed on Raman signals of cancerous breast tissue, and the authors show that it permits the identification of classification-relevant features, with the SVM yielding high accuracies in the reduced feature space. More importantly, the selected features are shown to be biologically relevant, emphasising the promise of the technique to select domain-adjusted features. Two previous studies conducted feature selection using the ant colony optimisation (ACO) algorithm to select Raman features [23,24]. The ACO algorithm is a swarm bionic intelligent optimisation algorithm is a wrapper-based algorithm, mimicking the methods by which ants create a path between their hill and a food source [24]. The algorithm has demonstrated robustness in past research and performed well in applications to Raman Spectroscopy signals. Ref. [23] used the ACO algorithm to select the most important features in the Raman signals of breast cancer. Using just five features, the classification accuracy was found to improve from the full dataset, reaching 87.7% in the multiclass classification. Ref. [24] identified five diagnostically relevant Raman bands using ACO in combination with an SVM classifier and reported an accuracy of 93.2% in the reduced feature space. These studies demonstrate that through targeted feature selection, the classification of Raman spectroscopy signals can be optimised, and explainable, biologically-relevant features can be extracted. One of the most common feature selection techniques is the use of model-assigned feature importance. Here, a classifier is trained on the complete spectrum and the importance assigned to each wavenumber during the training process is used to select the most important features. This is advantageous as the selected wavenumbers are adapted to the algorithm, potentially increasing its classification accuracy [25]. A summary of the retrieved studies applying feature selection for Raman spectroscopy data in the medical field is provided in Table 1.

Black-box models, including deep learning algorithms, have been implemented for diverse purposes in the Raman workflow [26,27]. Deep learning models offer a range of unique advantages, such as the ability to identify high-level patterns and extract unique spectral shapes, which recommends their deployment for feature selection [6]. However, the computation of feature importance for these models is more challenging due to their complex structure. Novel explainability methods offer one avenue to alleviate this constraint. By exploiting the algorithms’ mechanisms, these techniques are capable of computing feature importances for black-box deep learning models [21]. This study proposes the use of these feature weights for the implementation of a novel deep-learning-based feature selection method for Raman spectra. Two models, Convolutional Neural Networks (CNN) and Transformers, are chosen for this task. CNNs offer the advantage of shape recognition, which may improve the identification of important peaks. Transformers are advantageous due to their attention layers, which may choose several relevant spectral areas and hence identify correlated peaks. GradCAM and attention-head-based feature importance are used to compute feature weights for the two models, respectively, and features are selected on their basis. The performance of this feature selection methodology is compared to several established alternatives to assess the efficacy of the novel method.

As such, the main contributions of this research are as follows:

1. Two novel feature selection methods based on deep learning models are proposed for medical Raman spectroscopy.
2. The methods are evaluated across three medical datasets and compared to five established methods.
3. Recommendations for model and feature selection choices are made, considering sample size, task difficulty and level of data compression.

The rest of this report is structured as follows. Section 3 details the methodology of the study, including the selected classification and feature selection algorithms. The performance of these methods is reported and analysed in Section 4, and conclusions and recommendations for future research are provided in Section 5.

## 3. Materials and Methods

Three steps were taken to ascertain the performance of the novel feature selection methodology. First, four machine learning algorithms were deployed on the full spectrum of the data to compute baseline performance before feature reduction. Subsequently, seven feature selection techniques were implemented, including the two novel deep learning-based methods, and reduced datasets were computed. Finally, the four initial algorithms were deployed on the reduced datasets to assess performance based on different selection techniques. The two novel methods are presented alongside established techniques to allow for a frame of reference for their performance. Several degrees of feature reduction are computed to assess whether this impacts the performance of the selected techniques. Below, the four machine learning algorithms are first detailed in Section 3.1. Subsequently, the feature-selection methods are detailed in Section 3.2. For reproducibility, the code for all steps is available on GitHub https://www.github.com/ncrossberg/FeatureSelection (accessed on 13 August 2025). All models were optimised using Grid Search with a 5-fold cross-validation to select the best hyperparameters. The hyperparameter grids tested can be found in Table 2. The models were then retrained on the whole training set and tested on the reserved data. The full hyperparameter details for all models and datasets are available in the code. The details of the best-performing models in the full data are outlined below.

### 3.1. Model Implementation

Four models, an LDA, Random Forest, CNN and Transformer, were implemented and optimised for classification in each dataset. The models were selected based on a preliminary analysis of performance. Moreover, they were chosen to represent a spectrum of model complexity and learning paradigms, allowing for a comprehensive evaluation of the suitability of different model sizes. All models were trained and tested using a stratified 80–20 train-test split. A brief justification of the selection for each model is provided below. The key mathematical formulations of these models can be found in the Appendix A.

LDA was included due to its simplicity, interpretability, and efficiency, making it a useful baseline model for Raman spectroscopy. LDA is especially suitable for high-dimensional data with a high signal-to-noise ratio, making it suitable for Raman spectra, which tend to exhibit consistent peak shifts or intensities between classes.

Random Forest was chosen as a non-parametric ensemble method that is robust to noise and capable of modelling non-linear decision boundaries. It exhibits contrasting strengths to LDA and hence serves as an important comparator. It is especially useful for high-dimensional data, where relevant features may not be linearly separable.

CNNs were chosen for their ability to capture local spatial patterns as well as hierarchical features, which is especially valuable for Raman spectra, due to their characteristic peaks and localised signal features. CNNs are further capable of automatically learning filters that detect important spectral patterns without requiring handcrafted features.

The Transformer model was selected due to its ability to model long-range dependencies and contextual relationships. This is suitable for Raman spectra, where relevant features may not be strictly localised and may benefit from global context.

#### 3.1.1. Linear Discriminant Analysis

The LDA model uses a linear combination of selected features to distinguish between two or more classes. Class discrimination is enhanced by first identifying a subset of informative features and then projecting the original multidimensional data onto a lower-dimensional feature space. This dimensionality reduction simplifies the data structure, making the differences between classes more apparent. In the reduced feature space, an optical decision boundary is identified, which maximally separates the classes and minimises overlap. The model achieves this by maximising the between-class to within-class ratio of variance, ensuring that the classes are as distinct as possible [28]. This process makes LDA a robust method for classification and feature reduction [29]. The best-performing LDA model in this study consisted of an lsqr solver, automatic shrinkage, and a tolerance of 0.001.

#### 3.1.2. Random Forest

A Random Forest is an ensemble of decision trees. Decision trees are a non-parametric supervised machine learning algorithm which greedily search the feature space for a feature that can split the data while minimising entropy. The splitting process is repeated iteratively until a specified maximum tree depth is reached or the validation performance of the tree stops improving [30]. In a Random Forest, a specified number of decision trees are trained using bootstrapped samples of the training data, leading to varying structures between individual trees [31,32]. In highly dimensional data, the decision trees are trained on varying subsamples of the data, where more important features have a higher chance of being included in the subsamples and hence being assigned a higher importance value. The Random Forest classifies a given sample according to the majority vote produced by the individual decision trees. Random forests are frequently favoured over decision trees as they are considered to be more robust in their performance [33]. The hyperparameters of the best Random Forest model were 500 estimators, with entropy as the criterion and the square root for the maximum number of features.

#### 3.1.3. Convolutional Neural Network

A CNN is a sophisticated form of artificial neural network specifically designed for the analysis and interpretation of visual data. The key feature of a CNN is the use of convolutional kernels. These are a series of filters that search for given features in the input and mark their presence on a feature map. The convolutional kernel’s number, size and stride can be adjusted to improve model performance and avoid overfitting [34]. The feature maps are then summarised in a pooling layer to decrease dimensionality and increase processing speed. The number of convolutional and pooling layer stacks can be adapted depending on the input data, with larger numbers required for more complex data, allowing the extraction of high-level features [35]. The final feature maps are then flattened into a one-dimensional feature space before being passed through a dense layer, which classifies the input [36]. A CNN was chosen as a candidate model, as convolutional kernels may allow the identification of curve patterns and structures not prioritised by other models. In addition, this feature of the CNN may identify peaks and dips indicative of underlying biological chromophores, decreasing the distance between human and model decision processes. The CNN was implemented using Keras and TensorFlow Version 2.18 [37,38]. The best CNN model consisted of a convolutional layer with 16 filters, a kernel size of 12, and a step size of 10, followed by a MaxPooling layer, batch normalisation, a flattening layer, and two dense layers.

#### 3.1.4. Transformer

Transformer models are a type of neural network, initially introduced by [39], which are novel in their use of self-attention mechanisms. This enables them to weigh the importance of different elements of the input sequence. Through this, long-range dependencies and contextual nuances can be effectively captured [40]. A Transformer model was chosen for this analysis due to its usage of attention mechanisms, which may permit the identification of biologically relevant areas during classification. This would allow the construction of a model which makes decisions on the same basis as human experts. A traditional Transformer consists of an encoder and decoder block, each with multiple layers of self-attention and feed-forward neural networks. The encoder processes the input sequence to generate a rich contextual representation, which the decoder uses to produce the output sequence. Traditional Transformer models were designed for language processing tasks but can be adjusted to any one or multi-dimensional input [41]. The Transformer model in this study was implemented based on the vision Transformer proposed by [42] and was adapted for one-dimensional data. The best Transformer model in this study had a patch size of 15, a hidden size of 4, a depth of 3, 3 attention heads and a 4 MLP dimensions.

### 3.2. Feature Selection

This study aims to test the efficacy of the deep-learning-based feature selection methodology in comparison to established techniques for Raman spectroscopy data, which is both high-dimensional and multicollinear. To allow comparative analysis of the novel methods, five established feature-selection approaches were implemented in addition to the deep-learning-based approaches. All methods are presented below, alongside their advantages for Raman data. Model-based methods, including the novel deep-learning methods, are listed first, followed by three model-agnostic approaches: LinearSVC with L1 penalisation, select k-best and domain-based feature selection.

#### 3.2.1. LDA-Based Feature Selection

This feature selection method leverages feature importance values derived from the LDA model. During training, each feature is assigned a weight that reflects its ability to discriminate between classes by maximising separation in the feature space [29]. These weights serve as a proxy for feature importance, with higher values indicating greater relevance to the classification task. To select the most informative features, the weights are mean-aggregated and ranked accordingly. The features with the highest mean weights are selected to form a reduced feature set. This process decreases the data’s dimensionality while preserving the features most critical for classification, enabling models to be trained and evaluated in a streamlined feature space to assess their performance and efficiency.

LDA feature importance is suitable for the selection of Raman data due to its ability to handle the data’s multicollinearity. The construction of discriminant axes as linear combinations of the original variables allows reduced dependency on individual features, hence addressing multicollinearity [29]. The maximisation of between-class variance and minimisation of within-class variance ensures that the selected features meaningfully contribute to class separation, disregarding their interrelationships [43]. Moreover, the LDA’s dimensionality reduction capability preserves the most discriminative information, making it well-suited for extracting relevant features in complex datasets.

#### 3.2.2. Random Forest-Based Feature Selection

During the training of a Random Forest, each decision tree is built using a bootstrapped sample of the training data. At each split, a tree selects the features that result in the greatest reduction in impurity. This process is repeated for successive nodes, following the same principle [44]. Feature importance is determined by calculating the average impurity reduction contributed by each feature across all trees in the forest. These importances are computed separately for each class, revealing class-wise variations in feature usage. The importances are then mean-aggregated, allowing identification of the most important features.

Random Forest is selected, as it is highly robust to multicollinearity, decreasing the model’s reliance on correlated variables and hence increasing its utility for the selection of features in Raman data [45]. In addition, the computation of feature importance across all trees in the forest stabilises the feature rankings in the presence of multicollinearity, hence making the ranking robust to the effects of the data. Finally, Random Forest is capable of capturing complex interactions between features while identifying the most influential ones in the presence of multicollinearity. As such, Random Forest is suited for the selection of Raman data due to its robustness to multicollinearity, stability of feature importance and ability to capture complex feature interactions.

#### 3.2.3. CNN-Based Feature Selection

**Figure 2:**
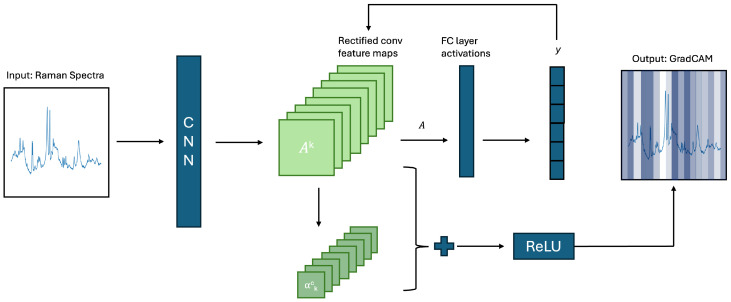
Schematic approximation of Grad-CAM mechanism.

The CNN’s feature importances were computed through the use of Grad-CAM, which is a local, model-based method, initially proposed by [46]. Grad-CAM computes the importance of feature bands based on the gradients flowing into the final convolutional layer of the CNN. Grad-CAM computes instance-wise feature importance, which is aggregated across all instances to understand total feature importance. A schematic representation of Grad-CAM can be seen in Figure 2. The figure represents the computed feature importances visually, with darker colours representing higher feature importance for the given wavenumber range. Note that this is a two-dimensional approximation of the current one-dimensional system. The schema is a re-drawing of the Figure used by [47]. Feature selection was conducted based on the Grad-CAM importances, and the features with the highest importance were retained.

GradCAM has previously been implemented to visualise feature importance in Raman data [48,49] and to select features in other fields [50,51,52]. However, no studies utilising it for feature selection in this domain were retrieved. The selection of features based on Grad-CAM computed feature importances offers several advantages for Raman spectroscopic data. The first is the ability of Grad-CAM to focus on informative regions of the spectrum. As a consequence, feature selection is conducted based on the features’ influence on the prediction rather than as a result of redundancies caused by multicollinearity. Second, the hierarchical feature extraction of CNNs allows the model to learn complex feature relationships, which may allow the identification of relevant features in the presence of strong correlations. Finally, the data-driven nature of CNN-based feature importance allows the model to learn data patterns independent of assumptions about data structure, making it adaptable to the unique structure of Raman spectroscopic data.

#### 3.2.4. Transformer-Based Feature Selection

The explanations for the Transformer model are extracted from the model’s attention-heads [53]. For each instance and attention-head, the self-attention value is extracted [42,54,55,56]. As the implemented algorithm consisted of three attention heads, this resulted in three attention values per feature and instance. The values were max-pooled, retaining the maximum attention value for each feature of a given instance. Max-pooling was chosen based on qualitative evaluation of each method’s visualisations. The feature importances were then aggregated across all instances to compute the average feature importance for the dataset. Features with the largest importance were selected and retained for further analysis. The Transformer-based feature selection offers the advantage of the attention mechanism, which may allow improved focus on relevant areas of the spectrum, leading to the identification of characteristic features.

The attention-head-based feature importance mechanism of the Transformer model is suitable for Raman spectroscopy data, for three key reasons. First, the context-aware feature selection of the model disentangles the contributions of correlated variables and focuses on relevant contextual interactions only, decreasing the impact of the multicollinearity of the data. Second, the attention mechanism of the model computes interactions between all pairs of input features, capturing global dependencies. Finally, the model’s attention mechanisms make it very robust to multicollinearity by prioritising features based on their direct contribution to the output, regardless of correlations.

#### 3.2.5. L1 Feature Selection

LinearSVC with L1 regularisation (referred to as L1 feature selection going forward) is a powerful method for feature selection in classification tasks. It is a variant of the SVM model that uses a linear decision boundary to separate data points of different classes. The key feature of L1 feature selection is the use of LASSO regularisation, which encourages sparsity in the model’s coefficients [57]. This sparsity means that the model will effectively shrink some of the feature weights to zero, effectively “selecting” a subset of features that contribute most to the classification decision [58]. Features with non-zero coefficients are considered important, while those with zero coefficients are discarded.

L1 feature selection regularisation was chosen due to its ability to effectively deal with high-dimensional datasets such as Raman spectroscopy data. The algorithm automatically performs feature reduction by setting the weights of less important or redundant features to zero, effectively excluding them from the model. This is beneficial in the presence of multicollinearity, as it helps to reduce the impact of correlated features that might otherwise introduce noise and destabilise model coefficients.

#### 3.2.6. K-Best Feature Selection

K-best feature selection with the ANOVA F-value evaluates each feature independently by calculating the ANOVA F-value, which quantifies the ratio of variance explained between groups to the variance within groups. A higher F-value indicates that a feature is more effective at discriminating between classes [59]. This method is particularly efficient and interpretable, making it suitable for high-dimensional datasets, including Raman spectroscopic data. By selecting the top-k features with the highest F-values, the approach focuses on those variables that contribute most significantly to the target variable, while remaining computationally lightweight.

K-best selection is included as a reference measure in the present study. The methodology yields certain advantages, including straightforward methods of feature selection, independent of other features, which ease its implementation. Additionally, the direct evaluation of the feature-target relationship allows an independent assessment of feature importance, regardless of multicollinearity [60]. However, this negligence may lead to the selection of multiple highly correlated features and hence the introduction of redundancy in the selected feature set. As such, this method is included as a comparison to the other selection techniques, which account for multicollinearity.

#### 3.2.7. Domain-Knowledge-Based Feature Selection

**Figure 3:**
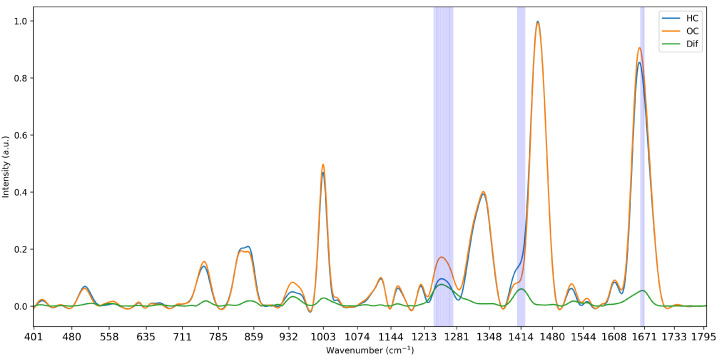
Visualisation of the mean intensity of the two classes of Dataset 1 and the computed difference in intensity and selected wavenumbers at 5% strictness.

The last feature selection method was based on the integration of domain knowledge into the selection pipeline. Here, the difference between the average intensity of all classes was computed to identify areas of maximum separation. In the case of multiple classes, the minimum reflectance was subtracted from the maximum reflectance of all classes for any given wavenumber. The absolute value of the differences was computed to compensate for any negative points, and the differences were sorted in descending order. Features were then selected based on these importance values, retaining wavenumbers at the points of largest separation. An illustration of the computation of differences can be seen in Figure 3. This methodology is tailored to the specifics of Raman data as it addresses the areas of separation between curves, which are caused by the underlying biological components of the measured tissues. While it therefore does not directly account for the multicollinearity of the data, it selects data-specific features, creating a representative subset of features.

### 3.3. Datasets

**Figure 4:**
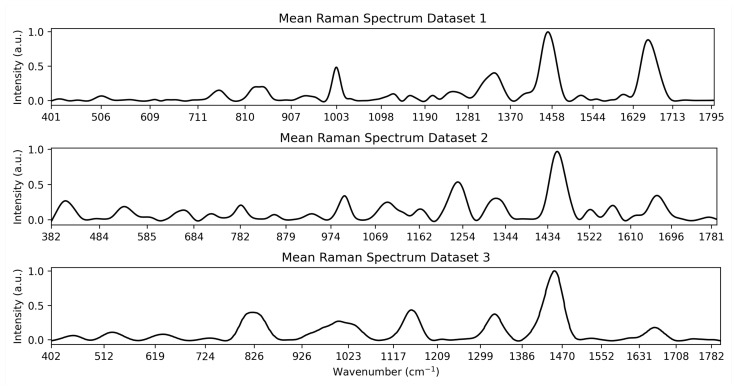
Visualisation of the mean spectrum for each Dataset.

This work makes use of three datasets to test the feature selection methodologies. All datasets are medical, and each is described in greater detail below. The datasets were selected for their diversity in application, size and number of outcome groups. The average curve for each Dataset is shown in Figure 4.

#### 3.3.1. Dataset 1

The first dataset [61] was collected by Paraskevaidi and Martin in 2018 [61]. A detailed account of data collection alongside the open-access dataset can be found in their paper “Raman spectroscopic techniques to detect ovarian cancer biomarkers in blood plasma” The dataset is available at ’Figshare’ under https://doi.org/10.6084/m9.figshare.6744206.v1. A summary of their collection methods is provided below. A total of 374 spectral measurements were collected, with 189 stemming from subjects with ovarian cancer and 185 from control subjects. Ovarian cancer is a serious health risk as the deadliest gynaecological malignancy, and optical methods, including Raman spectroscopy, provide an important opportunity for non-invasive detection [62]. Blood serum samples were collected from 27 women with ovarian cancer (OC) and 28 control subjects (HC), including healthy female volunteers as well as women with prolapse or benign gynaecological conditions. Blood was collected in EDTA tubes and centrifuged to remove cells from the plasma. The supernatant was then extracted and stored at −80 °C and thawed at room temperature before the collection of spectroscopic signals. The Raman spectra were collected using an InVia Renishaw Raman spectrometer coupled with a charge-coupled device (CCD) detector and a Leica microscope. Signals were collected in the wavenumber range of 1800.5–401.2 cm^−1^.

Before the implementation of machine learning models, the signals were pre-processed to denoise, baseline correct, and normalise the data. All three steps were implemented using the Raman Spy library [63]. Each step is described in turn below:

1. **De-noising**: Whittaker-Henderson smoothing was implemented to de-noise the data. This employs a discrete, penalised least-squares algorithm.
2. **Baseline Correction**: Doubly re-weighted penalised least squares was implemented to baseline correct the data. This helps counter the fluorescence effect, which causes peak shifts and may lead to model overfitting [64].
3. **Data Normalisation**: Max Intensity scaling was used to normalise the scale of the spectra.

#### 3.3.2. Dataset 2

The second dataset [65] was collected by Ho et al. [65] in 2019 and is composed of the Raman signals of five types of bacterial species The full ’Bacteria-ID’ dataset is available on GitHub at https://www.github.com/csho33/bacteria-ID (accessed on 13 August 2025). A total of 12,500 Raman spectra of five types of bacteria are recorded, with 10,000 spectra included in the training set and the remaining 2500 reserved for testing. All details regarding instrumentation and measurement procedure can be found in the original paper by [65]. The spectra are preprocessed using the same methodology described in Section 3.3.1, implementing Whittaker Henderson denoising, baseline correction via doubly re-weighted penalised least squares and MaxIntensity Data normalisation.

#### 3.3.3. Dataset 3

The third dataset [9] was collected by Yin et al. [9] in 2021 and published as part of their paper “An efficient primary screening of COVID-19 by serum Raman spectroscopy” The data is freely available on ’Figshare’: https://doi.org/10.6084/m9.figshare.12159924.v1. The data consists of the Raman signals of serum collected from patients belonging to three classes: healthy, suspected COVID-19 and diagnosed with COVID-19. Data was collected from a total of 177 patients, and a total of 465 instances were retained. The dataset was balanced with 159 COVID-19 positive spectra, 156 COVID-19 suspected spectra and 150 healthy control spectra. The data was collected by centrifuging blood samples at 3000 rpm for ten minutes, after which the samples were stored at 4 °C and measured within thirty-six hours of the time of collection. All details regarding instrumentation and measurement procedure can be found in the original paper by [9].

### 3.4. Sample Size

The three datasets vary considerably in sample size, and it is important to take the effect of this into consideration when evaluating their performance. Dataset 2, with 10,000 spectra in the training set, is by far the largest dataset, which gives it greater potential for performance as more data is available for pattern extraction during the training phase and decreases the likelihood of overfitting. However, the dataset also has the largest number of classes, increasing the difficulty of the task. Datasets 1 and 3 are comparable in size, with Dataset 3 having a slightly larger sample but also conducting multi-class classification, which is generally more challenging than the binary task for Dataset 1. It is important to note that comparisons of accuracy can only be made within a given dataset, due to variations in data type, collection procedure, and the type and difficulty of the classification. While the above guidelines regarding the effects of sample size and classification task generally hold, it is important to consider that variations in tissue structure and distinctiveness of different classes may affect the outcome.

## 4. Results and Discussion

**Figure 5:**
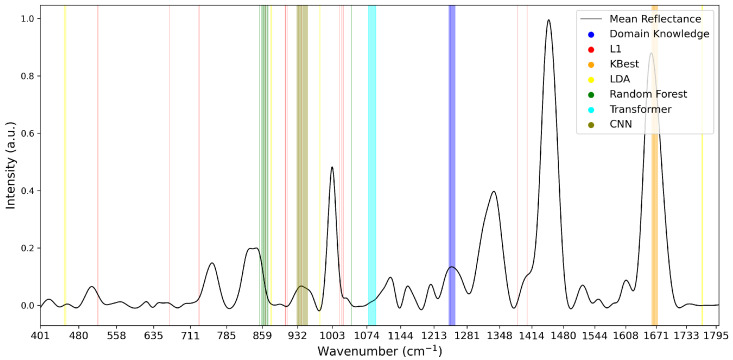
Selected Features at 1% for Dataset 1.

**Figure 6:**
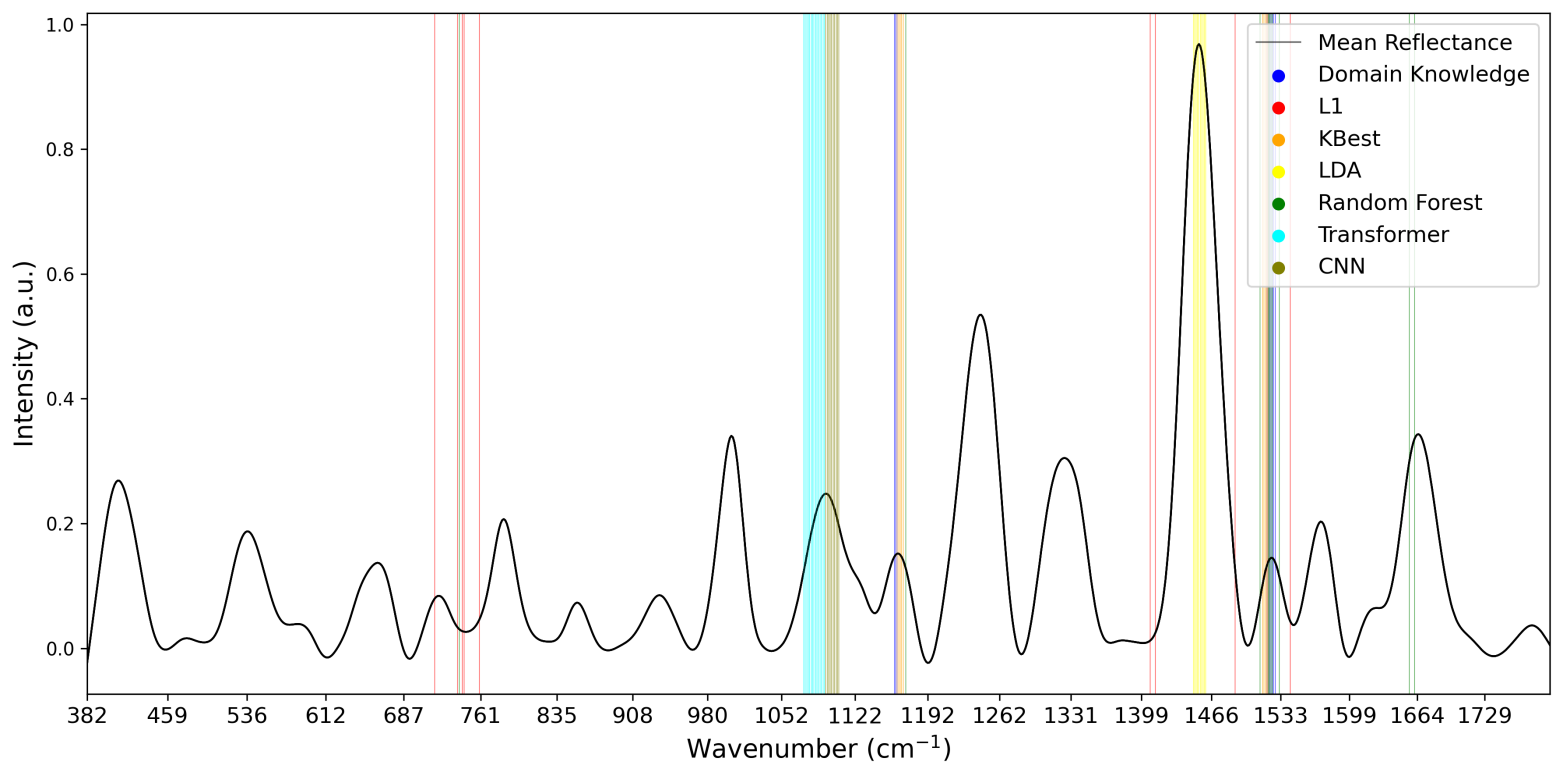
Selected Features at 1% for Dataset 2.

**Figure 7:**
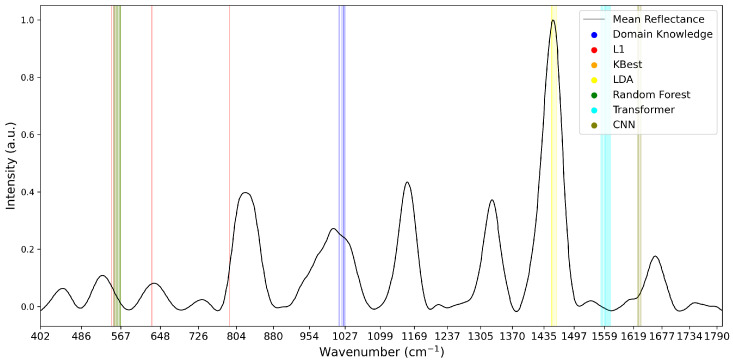
Selected Features at 1% for Dataset 3. Note that KBest and Random Forest features overlap at 553–567 cm^−1^, impacting visibility.

To analyse the variations of feature selection methodologies, the selected features at 1% retention for each technique and each dataset are visualised in Figure 5, Figure 6 and Figure 7. This emphasises the considerable difference in selected features between techniques, highlighting the importance of elucidating the variations in classification success between techniques. Interestingly, the majority of methods appear to cluster their feature importance. An exception to this is the L1 feature selection, which makes sense because L1 penalises the absolute size of coefficients. As such, if there are nearby correlated wavenumbers, it will select one or a few of the features and skip the remainder, leading to the distribution observed in the figures. While the chosen wavenumber ranges generally vary between the techniques, the CNN, K-best and L1 all cluster in dataset 3 (Figure 7). This may indicate the presence of important features at these wavenumbers, as multiple selection techniques converge. The patterns in feature selection for each methodology appear to vary considerably between the datasets. For example, while the LDA selects large peaks in datasets 2 and 3, it focuses on the beginning and end of the spectrum in dataset 1. Additionally, while the CNN selects smaller but interestingly-shaped peaks in datasets 1 and 2, it considers the rising and sinking curve shapes important in dataset 3. One cause for these differences may be considerable variations in the curve shapes of the datasets, leading to the selected features being significant for some spectra but not displaying these patterns on the plotted mean curve.

The remainder of this paper will first detail the model performance on the full dataset, analysing classification accuracy and proposing possible reasons for discrepancies in classifier performance. The results of the feature selection analysis are then presented, and trends in the selection and classifier methodology are analysed. Possible reasons for these patterns in behaviour are postulated. Finally, Section 5 summarises the findings and provides recommendations for future research.

### 4.1. Full Datasets

To establish the baseline performance of each model, the classifiers were trained and optimised on the full spectral ranges. The top row in Table 3 shows the performance of the four models in each dataset before feature selection. Across all three datasets, the CNN is found to perform best when classifying the full range of data, most likely due to the model’s ability to extract significant curve shapes and effectively reduce input dimensionality. The Transformer performs remarkably poorly across all three datasets. This is likely due to the model’s complexity and consequential tendency to overfit, which is exacerbated by the relatively small size of the datasets. Upon inspection, the Transformer exhibits significantly higher training-set accuracies, confirming the overfitting hypothesis. While the model size was adjusted during implementation to decrease complexity and simplify its structure, overfitting can not be entirely prevented due to the general size of Transformer models. However, despite these problems, Transformers were retained for analysis, as their feature extraction methods via attention heads may allow the identification of important parts of the spectrum.

The Random Forest performs comparatively poorly in the full dataset. This is interesting, as its performance increases significantly after feature selection, which is especially noteworthy as it is expected to be more robust to highly collinear data than the LDA. One reason for this may be that Random Forest could be more prone to overfitting compared to LDA. Additionally, as a non-parametric model, Random Forest may struggle if non-relevant features dominate or its feature selection during bootstrapping does not function well. The LDA, on the other hand, performs comparatively well, exhibiting accuracies on par with the CNN. This highlights the model’s ability to draw linear separations between classes despite the multicollinearity of the data.

### 4.2. Feature Selection Results

A summary of the results of the classification after feature selection is shown in Table 3. The best accuracy per classifier is highlighted in bold. A more comprehensive display of results, including averages across models and datasets, can be found on GitHub https://www.github.com/ncrossberg/FeatureSelection (accessed on 13 August 2025). Overall, the classification accuracies remain very high in a constraint feature space with the implemented models achieving accuracies of 80%, 86% and 74% in the three datasets, respectively, while only retaining 5% of the features. Furthermore, accuracies on par with those achieved in the full dataset are observed when retaining only 10% of the data and using either Random Forest or CNN-based feature selection algorithms. This highlights the efficacy of these methods in extracting representative features from highly dimensional and multicollinear data. The performance of all classifiers decreases considerably in the 1% feature space, falling by over 15% for all three datasets. This is attributed to a loss of representation of the complex relationships previously captured in the larger feature spaces. The L1’s improved performance in the reduced feature space may also be attributed to the spread of considered wavenumbers, extracting information from several sections of the spectrum rather than focusing on a single part of the curve.

No significant differences in accuracy are found between scenarios where features are selected and classified by the same model as opposed to a different model. As such, it may be inferred that features selected by a given algorithm’s feature importance are not necessarily more suitable for classification by the given algorithm. Overall, it appears that features extracted by the Random Forest or CNN perform best for the majority of models, with 41 out of 60 of the best classification performances per model and dataset stemming from these feature selection algorithms. This may be due to the method by which the algorithms assign feature importance. The Random Forest’s averaging of importance across multiple trees designates high relevance to consistently well-performing features, which may make feature selection on its basis more robust compared to other techniques. Additionally, when dealing with high-dimensional data, Random Forests select a subset of features during each iteration of bootstrapping. This increases the likelihood of a truly important feature being selected over random noise, hence improving the Random Forest’s ability to identify relevant features and generate robust feature subsets. Several aspects of the CNN’s structure may lead to its success as a feature selection technique. Its ability to recognise local patterns makes it adept at identifying spectral signatures and extracting consequently relevant features. This is especially important as CNNs are capable of identifying important spectra shifts in the presence of the noise inherent to experimental variations during Raman signal collection. These structural aspects may make the models especially adept at selecting relevant features and hence lead to their superior performance.

Overall, the two proposed deep learning-based techniques differ considerably in their performance, emphasising that deep learning-based feature selection as a whole is not a guarantee for success. However, the features selected by the CNN function reasonably well, emphasising its promise in the domain. A potential reason for the poor performance of the Transformer is related to its poor baseline performance, indicating that even in the full dataset, the model is not capable of identifying classification-relevant features. This may be due to the significant complexity of the Transformer algorithm, which requires large datasets to perform effectively. This is challenging in the medical domain, as ethical constraints surrounding medical data collection make it challenging to build datasets large enough to effectively train such models.

Interestingly, when retaining 5–20% of features, the model-based feature selection algorithms consistently outperform K-best, L1 and domain-knowledge-based selection, with the CNN and Random forest performing especially well, yielding the best accuracy in 13 out of 15 cases. However, at the 1% level, these techniques drop in performance, with the L1 and K-best methodology now yielding the best results. One possible explanation for this is that the features selected by the model-based algorithms function well in conjunction with each other due to capturing relationships between variables identified through the respective classification algorithms. However, these relationships are lost as the number of features decreases further, leading to a drop in performance. The L1 and K-best algorithms may perform better since they select independent features rather than capturing relationships. As compression increases, these features retain importance while the relationships captured by the model-based techniques may be lost. This may account for the improved performance of the L1 and K-best selection algorithms.

Domain-knowledge-based feature selection is found to perform poorly at all levels. One possible reason for this is that features are selected based on mean aggregations of the individual instances without accounting for intra-class variance. As such, differences between classes present at the mean level may not be consistent across instances, leading to the selection of non-representative features. Another possible reason may be that domain-knowledge-based selection is incompatible with data-driven classification, as the patterns identified based on human intuition are not in line with the processing of the machine learning algorithms. Hence, data-driven feature selection may be more suitable as the selected wavenumbers are consistent with the processing mechanisms of the implemented classifiers.

The best classifiers in the reduced feature space are the Random Forest and the CNN, achieving the highest classification accuracy in six and eight out of fifteen cases, respectively. The suitability of the Random Forest may be attributed to the structure of the model. Through feature subsampling during tree construction, the model relies on a feature subset by design, making it less reliant on the full feature set and more suitable for classification after feature selection. Additionally, the ensemble nature of the model may compensate for the reduced variance in a smaller feature set, as the ensemble effect may compensate for information loss. The CNN’s high performance may be related to the noise reduction associated with feature selection, which allows for more efficient extraction of patterns by the algorithm. Additionally, the feature reduction may mitigate the overfitting tendencies of the CNN, resulting in improved performance.

It is important to note that classifier performance varies with sample size across the different datasets. While the Transformer model has a tendency to overfit in most trials, this effect is notably less severe in Dataset 2. As shown in Table 3, the Transformer demonstrates a relatively strong performance in this dataset, ranking highest at the 1% feature level. This suggests that, although the model does not consistently generate high-quality features (as evidenced by only 2 of the top-performing models being based on Transformer-derived features), it can still perform reasonably well as a classifier when applied to a sufficiently large dataset. Therefore, future research may wish to explore using the Transformer as a classifier in combination with alternative feature extraction methods, particularly for larger Raman datasets.

Based on these results, it can be seen that while no single feature selection method is optimal, several trends can be identified and recommendations are made on their basis. Overall, Random Forest and CNN are found to perform best for both feature selection and classification when moderate levels of compression are required. As such, these models may function as a suitable baseline for future studies. For larger datasets, the Transformers may work well as classifiers, especially if stark compression is required. This may function well in conjunction with either L1 or Random Forest-based feature selection. L1 feature selection is recommended for cases where strong compression is required, but the best classifier for the compressed feature space may vary on a case-by-case basis. In general, it is recommended to consider the dataset size, with less data requiring smaller models, the level of compression and the difficulty of the task, with more difficult tasks requiring more complex models.

## 5. Conclusions

This study evaluated several feature selection methods for Raman spectroscopy data to improve the implementation of explainability techniques. Two novel methods for feature selection based on explainable deep-learning models were implemented and compared to established techniques. Generally, models using only 10% of the original features achieved accuracy levels comparable to those trained on the full spectra. Model-based feature selection, especially using Random Forest and CNN, yielded the best overall performance. However, when reducing the feature set to just 1%, L1 outperformed the other approaches. No significant advantage was observed when the same model was used for both feature selection and classification. The novel feature selection approach using CNN in conjunction with GradCAM has the highest accuracy on average, although no single approach consistently outperformed all others across scenarios. While no definitive recommendations can be made, some general patterns emerged. If stark compression of the Raman spectrum is required, statistical techniques perform best. If more features can be maintained, machine learning-based feature selection is recommended. The selection of features on a domain-knowledge basis was not found to perform well in this study, but other operationalisations of this technique should be explored.

## Figures and Tables

**Table 1:** Overview Table of the Literature applying Feature Selection for Medical Raman Spectroscopy Data.

Citation	Feature Selection Method	Advantage	Machine Learning	Deep Learning
Wei et al. (2023) [21]	Long-Short Term Memory Model	Long-term dependencies	x	x
Fenn et al. (2013) [22]	Fisher-based feature selection	Reduced overfitting		x
Fallahzadeh et al. (2018) [23]	Ant Colony Optimisation	Robust and combinable	x	
Li et al. (2014) [24]	Ant Colony Optimisation	Robust and combinable	x	
Romanishkin et al. (2022) [19]	Fisher Criterion	Reduced overfitting	x	
Plante et al. (2021) [20]	SVM and Gaussian	Local and long-range features	x	

**Table 2:** The Hyperparameters used to tune the networks during cross-validation in the training set.

Algorithm	Hyperparameter	Lower	Upper	Other
LDA	Solver			svd, lsqr, eigen
	Shrinkage	0.1	1	
	Tolerance	0.001	0.00001	
Random Forest	n estimators	50	600	
	criterion			gini, entropy, log loss
	max depth	5	20	None
	min samples split	1	10	
	min samples leaf	1	10	
CNN	optimiser	0.01	0.0001	Adam, Adamax
	conv filters	8	64	
	conv window	8	14	
	conv stride	5	14	
	pool size	2	8	
	pool stride	2	8	
	batch momentum	0.70	0.90	
	batch epsilon	0.01	0.0001	
	dense size	128	1024	
Transformer	patch size	2	30	
	hidden size	2	256	
	depth	3	6	
	num heads	2	6	
	mlp dim	2	128	
	sd survival probability	0.8	1	

**Table 3:** Classification Accuracy (%) for the full dataset and different levels of feature selection.

		Dataset 1				Dataset 2				Dataset 3			
		LDA	RF	CNN	Trans	LDA	RF	CNN	Trans	LDA	RF	CNN	Trans
Full Data		0.84	0.80	**0.86**	0.57	0.91	0.88	**0.94**	0.64	**0.81**	0.77	**0.81**	0.59
20%	LDA	0.72	0.64	0.65	0.49	0.87	0.84	0.91	**0.86**	0.73	**0.80**	0.71	0.34
	RF	0.73	0.63	0.66	0.57	**0.89**	**0.87**	**0.93**	0.90	0.74	0.77	**0.80**	0.32
	CNN	**0.79**	**0.82**	0.83	**0.64**	0.86	0.85	0.91	0.84	**0.77**	0.78	0.77	0.46
	Trans	0.71	0.79	**0.85**	0.49	0.77	0.78	0.82	0.78	0.61	0.70	0.73	**0.51**
	L1	0.69	0.71	0.67	0.50	0.69	0.59	0.73	0.75	0.58	0.6	0.59	0.34
	K-Best	0.63	0.67	0.66	0.59	0.50	0.46	0.53	0.49	0.61	0.61	0.58	**0.51**
	Domain	0.76	0.76	0.71	0.59	0.78	0.79	0.83	0.77	0.68	0.65	0.66	0.54
15%	LDA	0.71	0.62	0.69	0.50	0.84	0.80	0.90	0.83	0.72	0.76	0.65	0.33
	RF	0.74	0.68	0.72	**0.63**	**0.87**	**0.86**	**0.92**	**0.87**	0.74	0.77	**0.75**	0.37
	CNN	**0.77**	**0.83**	**0.82**	0.49	0.82	0.82	0.87	0.83	**0.78**	**0.80**	0.74	0.42
	Trans	0.73	0.63	0.78	0.59	0.79	0.80	0.84	0.78	0.49	0.69	0.62	0.33
	L1	0.69	0.74	0.61	0.50	0.69	0.61	0.73	0.76	0.58	0.63	0.61	0.33
	K-Best	0.63	0.66	0.59	0.59	0.50	0.46	0.53	0.49	0.61	0.58	0.56	**0.55**
	Domain	0.77	0.74	0.64	0.59	0.78	0.77	0.81	0.76	0.60	0.65	0.70	0.55
10%	LDA	0.69	0.64	**0.78**	0.50	0.78	0.76	0.85	0.76	0.66	**0.80**	0.65	0.34
	RF	0.69	0.65	0.74	0.50	**0.86**	**0.85**	**0.91**	**0.85**	**0.70**	**0.80**	**0.68**	0.44
	CNN	**0.75**	**0.86**	0.75	0.51	0.81	0.81	0.86	0.78	**0.70**	0.72	**0.68**	0.45
	Trans	0.71	0.54	0.60	0.50	0.72	0.75	0.78	0.74	0.57	0.61	**0.68**	0.34
	L1	0.69	0.67	0.59	0.50	0.69	0.6	0.74	0.77	0.58	0.60	0.61	0.33
	K-Best	0.63	0.69	0.68	0.59	0.50	0.46	0.53	0.49	0.61	0.60	0.57	**0.59**
	Domain	0.70	0.70	0.58	**0.61**	0.69	0.72	0.75	0.69	0.59	0.58	0.60	0.60
5%	LDA	0.71	**0.76**	0.66	0.50	0.66	0.67	0.74	0.65	0.63	0.71	0.60	0.34
	RF	0.66	0.62	0.65	0.50	**0.81**	**0.81**	**0.86**	**0.82**	**0.66**	0.72	0.70	0.52
	CNN	**0.76**	0.75	**0.80**	0.50	0.70	0.73	0.77	0.70	0.65	**0.74**	**0.72**	0.34
	Trans	0.70	0.63	0.66	0.50	0.66	0.68	0.71	0.66	0.61	0.60	0.63	0.34
	L1	0.69	0.69	0.63	0.50	0.69	0.60	0.74	0.75	0.58	0.61	0.62	0.55
	K-Best	0.63	0.66	0.61	0.59	0.50	0.47	0.53	0.51	0.61	0.62	0.56	**0.57**
	Domain	0.53	0.63	0.57	**0.62**	0.64	0.64	0.66	0.63	0.46	0.54	0.51	0.46
1%	LDA	0.67	0.60	0.65	0.50	0.41	0.39	0.44	0.42	0.49	0.51	0.34	0.33
	RF	0.61	0.57	0.63	0.39	0.65	**0.70**	0.71	0.69	0.59	0.39	0.59	**0.57**
	CNN	0.62	0.63	**0.70**	**0.59**	0.35	0.34	0.41	0.38	0.33	0.39	0.31	0.34
	Trans	0.51	0.51	0.52	0.50	0.34	0.32	0.39	0.33	0.46	0.48	0.46	0.33
	L1	**0.69**	**0.70**	0.62	0.50	**0.69**	0.60	**0.73**	**0.75**	0.58	**0.65**	**0.62**	0.52
	K-Best	0.63	0.64	0.59	**0.59**	0.50	0.47	0.53	0.49	**0.61**	0.62	0.56	0.34
	Domain	0.62	0.64	0.61	0.58	0.50	0.48	0.53	0.51	0.39	0.47	0.53	0.41

## Data Availability

These data were derived from the following resources available in the public domain: Dataset 1–Figshare: https://doi.org/10.6084/m9.figshare.6744206.v1, Dataset 2–GitHub: https://github.com/csho33/bacteria-ID (accessed on 13 August 2025), Dataset 3–Figshare: https://doi.org/10.6084/m9.figshare.12159924.v1.

## Abbreviations

The following abbreviations are used in this manuscript:

ANOVA	Analysis of Variance
CNN	Convolutional Neural Network
Grad-CAM	Gradient-weighted Class Activation Mapping
LDA	Linear Discriminant Analysis
RF	Random Forest
Trans	Transformer

## Appendix A

The following presents the key mathematical formulas for each of the four implemented classification models.

### Appendix A.1. LDA

For the LDA model, the linear discriminant function $\delta_{k}\left(x\right)$ for class *k* is given by:

$$\delta_{k}\left(x\right)=x^{T}\Sigma^{-1}\mu_{k}-0.5\mu_{k}^{T}\Sigma^{-1}\mu_{k}+log\left(p_{k}\right)$$

This means that for a given input *x*, the function assigns it to the class with the highest value of $\delta_{k}\left(x\right)$.

### Appendix A.2. Random Forest

For a Random Forest, where the training set *D* is denoted by

$$D=\{\left(x_{1},y_{1}\right),\left(x_{2},y_{2}\right),...,\left(x_{N},y_{N}\right)\}$$

The final prediction is computed using

$$\hat{y}=mode\left\{h_{1}\left(x\right),h_{2}\left(x\right),...,h_{M}\left(x\right)\right\}$$

where $\hat{y}$ is the final predicted class, $h_{k}\left(x\right)$ is the class assigned by the $k^{th}$ decision tree for a given input *x* and mode aggregates the individual predictions, returning the most frequent class.

### Appendix A.3. CNN

For the CNN, the convolutional layer is computed as

$$S\left(i\right)=\left(I*K\right)\left(i\right)=\sum_{m}I\left(i-m\right)*K\left(m\right)$$

where *I* is the input sequence, *K* is the 1D kernel or filter vector and *S* is the output feature sequence.

The Max Pooling layer is computed as

$$P\left(i\right)=\underset{m\in window}{max}S\left(i\right)$$

where *S* is the input feature sequence and *P* is the output pooled feature sequence.

### Appendix A.4. Transformer

For the transformer, the multi-head attention is computed using

$$MultiHead\left(Q,K,V\right)=Concat\left(head_{1},...,head_{h}\right)W^{O}$$

where each head is computed as:

$$Attention(QW_{i}^{Q},KW_{i}^{K},VW_{i}^{V})$$

where $W^{O}$ is the final linear transformation weight matrix, $W_{i}^{Q},W_{i}^{K},W_{i}^{V}$ are learnable weight matrices for each head, and Attention is computed using:

$$Attention\left(Q,K,V\right)=softmax\left(\frac{QK^{T}}{\sqrt{d_{k}}}\right)V$$

where Q,K,V are matrices representing the queries, keys, and values and $d_{k}$ are the dimensions of the key vectors, used for scaling to prevent the dot product from becoming too large.
